# Utilization of whole-body computed tomography in trauma: The role of trauma center level and ACS verification

**DOI:** 10.1016/j.sopen.2026.08.003

**Published:** 2026-09-02

**Authors:** Daniel Tabibian, Jeffrey Balian, Kevin Tabibian, Arjun Chaturvedi, Barzin Badiee, Troy Coaston, Nam Yong Cho, Areti Tillou, Peyman Benharash

**Affiliations:** a Center for Advanced Surgical and Interventional Technology (CASIT), David Geffen School of Medicine at University of California, Los Angeles, CA, USA; b Division of Trauma & Acute Care Surgery, Department of Surgery, David Geffen School of Medicine at University of California, Los Angeles, CA, USA; c Department of Surgery, David Geffen School of Medicine at University of California, Los Angeles, CA, USA

**Keywords:** Whole-body computed tomography, Selective computed tomography, Trauma Quality Improvement Program, Motor vehicle collision, American College of Surgeons Committee on Trauma verification

## Abstract

**Background:**

Whole-body computed tomography (WBCT) is frequently used in trauma, though its value in stable patients remains uncertain. We examined WBCT use after motor vehicle collision (MVC) and whether trauma center designation and American College of Surgeons Committee on Trauma (ACS-COT) verification were associated with imaging practices.

**Methods:**

Using 2018–2021 Trauma Quality Improvement Program (TQIP) data, we identified adults aged 18–65 with blunt MVC who were stable (systolic blood pressure ≥ 100 and Glasgow Coma Scale 15) and received a CT scan. WBCT was defined as head, chest, and abdomen CT within 2 h of admission; selective CT (SCT) covered fewer than all three regions. Temporal trends by state-designated trauma level and ACS-COT verification were assessed using Cuzick's nonparametric test, and multivariable logistic regression identified factors associated with WBCT use.

**Results:**

Among 288,264 stable MVC patients receiving CT, 78,796 (27.3%) underwent WBCT, and 209,468 (72.7%) SCT. WBCT utilization declined at ACS-verified (31.9 to 30.2%) and non-verified centers (22.0 to 18.4%), and at Level I (29.1 to 27.1%) and Level II (28.5 to 26.9%) facilities (all nptrend <0.001), with consistent declines across Injury Severity Score strata. After adjustment, ACS verification was associated with higher odds of WBCT (AOR 1.96, 95% CI 1.92–2.00), whereas non-profit or government ownership (AOR 0.55, 95% CI 0.54–0.57) was associated with lower odds.

**Conclusions:**

In stable MVC patients, WBCT utilization declined from 2018 to 2021 across trauma levels and verification strata, while institutional characteristics remained strongly associated with WBCT.

## Introduction

Globally, injuries from trauma result in approximately 4.4 million deaths annually, comprising nearly 8% of all mortality [1]. Patients admitted for trauma may present with multisystem and occult injuries, prompting physicians to perform a series of diagnostics during the initial phases of treatment. Computed tomography (CT) is a commonly utilized imaging modality in trauma, favored for its rapid acquisition and high diagnostic sensitivity [2]. While technical advances in x-ray sources and image processing have allowed for the wide availability of whole-body CT (WBCT), the role of this modality in hemodynamically stable patients remains controversial [3], [4].

Prior studies have demonstrated WBCT to enhance detection of occult injuries, improve diagnostic accuracy, and increase survival rates compared with selective CT (SCT) [5]. However, the recent REACT-2 clinical trial reported no survival benefit with WBCT compared to selective regional imaging [3]. Other work has highlighted potential drawbacks of WBCT, which include increased radiation exposure and a higher frequency of incidental findings, factors that may delay definitive treatment and elevate patient anxiety [6], [7]. These findings underscore ongoing uncertainty regarding the optimal imaging strategy in trauma care.

Earlier investigations have documented a general rise in WBCT use across the United States [8]. Bunn and colleagues previously reported rising WBCT use among stable MVC patients through 2016, with greater utilization at Level II than at Level I trauma centers [17]. Whether this trend has persisted into the contemporary era is unclear, as are the institutional factors underlying it. In light of advances in CT technology and evolving clinical protocols, further assessment of WBCT utilization trends is warranted. The present study utilized a nationally representative cohort to characterize contemporary trends in WBCT utilization among hemodynamically and neurologically stable patients following motor vehicle collisions (MVC). Centers were stratified by state-designated trauma level and American College of Surgeons Committee on Trauma (ACS-COT) verification status to evaluate whether institutional characteristics and accreditation standards were associated with imaging practice patterns. We hypothesized that institutional characteristics, including ACS-COT verification status and hospital ownership, would be independently associated with the odds of receiving WBCT. We further hypothesized that proportional WBCT utilization would shift over the study period as trauma imaging practice continued to evolve.

## Methods

This was a retrospective cohort study utilizing the 2018–2021 Trauma Quality Improvement Program (TQIP) participant use files. Using a validated data management system, TQIP reports clinical data from more than 900 trauma centers across the United States [9]. TQIP submission is required for ACS-COT verified centers as a condition of verification but voluntary for non-verified state-designated trauma centers. Both groups are represented in the participant use file and were identified by the ACS-COT verification status reported by TQIP. This study was deemed exempt from full review by the institutional review board at the University of California, Los Angeles.

**Fig. 1:**
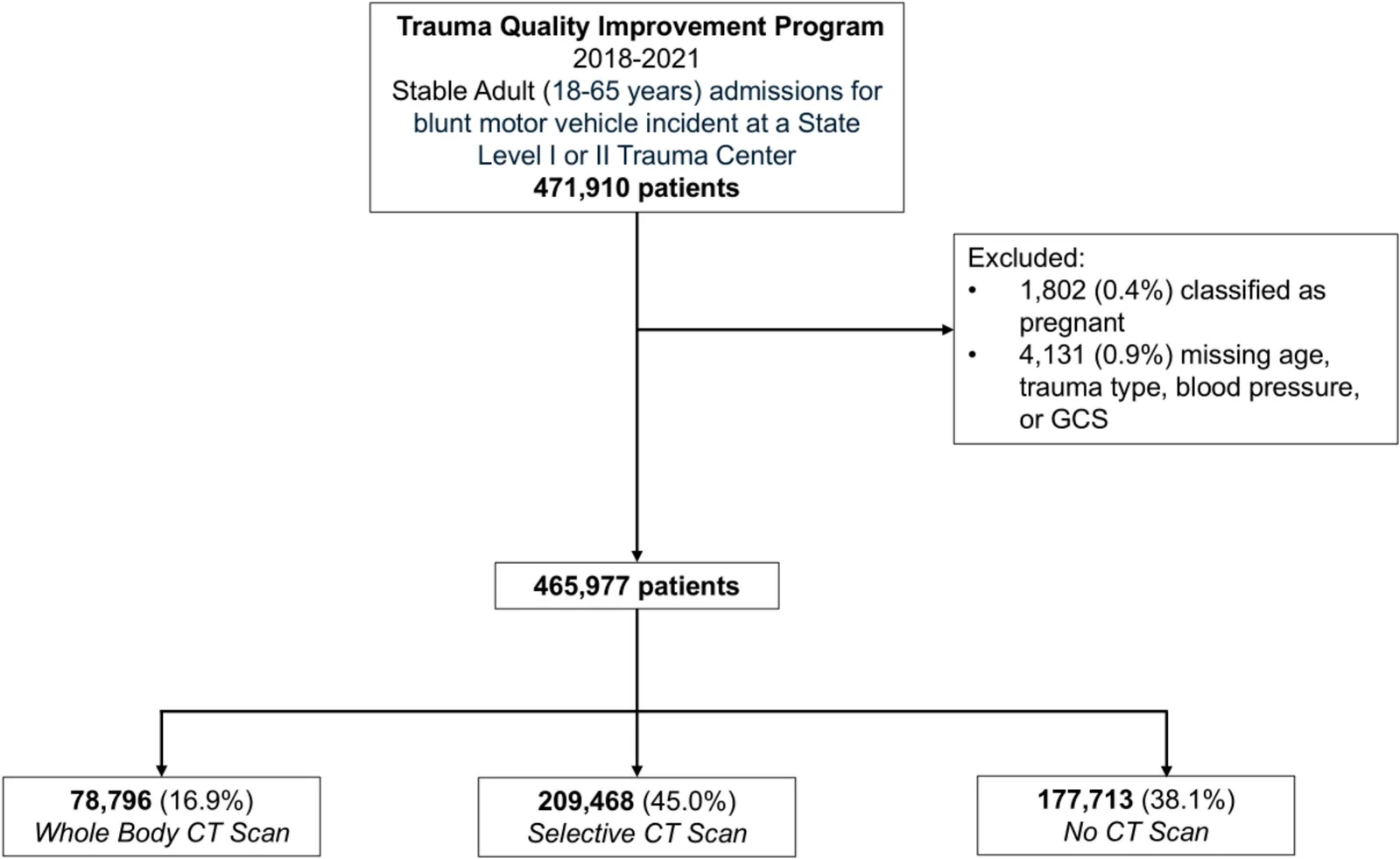
CONSORT flow diagram of patient inclusion and CT imaging classification; CT, computed tomography.

Records for adult (18–65 years) patients presenting with blunt trauma after motor vehicle crash (MVC), were identified. Only neurologically and hemodynamically stable subjects, defined as having a Glasgow Coma Scale Score (GCS) of 15 and systolic blood pressure (SBP) ≥ 100, were considered. The SBP ≥100 mmHg threshold was selected as a widely used cutoff for hemodynamic stability in trauma evaluation. Patients older than 65 years were excluded because this age group is conventionally classified as geriatric and carries a distinct comorbidity and frailty burden, with registry-based analyses showing steep increases in trauma mortality across the older age range that would confound the present analysis [10], [11]. Records missing key data such as age, trauma type, systolic blood pressure, or GCS, were also excluded (Fig. 1). We also did not consider patients who were determined to be pregnant based on the TQIP comorbidity indicator. Whole-body computed tomography was defined as having a head CT along with a simultaneous chest and abdomen CT. Selective computed tomography was defined as having a CT scan of at least one of the head, chest and abdomen, but not all regions. Whole-body and selective CT scans performed within 2 h of admission were considered. Pelvic and cervical spine CT were not required as separate components of this definition. Abdominal and pelvic imaging are typically acquired together in trauma evaluation and were captured under the abdominal CT designation, and dedicated procedure codes for pelvic and cervical spine CT are inconsistently recorded in the registry; incorporating them produced whole-body CT rates well below those reported in comparable national work. The definition was therefore restricted to head, chest, and abdominal acquisition, consistent with a prior Trauma Quality Improvement Program analysis that applied the same regional definition [17]. WBCT utilization was defined as the proportion of patients receiving WBCT among those who underwent any CT imaging. We evaluated incidence and utilization of WBCT and SCT based on state-designated level I and II trauma centers as well as by the American College of Surgeons Committee on Trauma (ACS-COT) verification status. Hospital type was obtained from TQIP and categorized as for-profit, non-profit, or government using the TQIP teaching status and ownership variables, consistent with TQIP data dictionary definitions. Demographic, insurance, vehicle safety (seatbelt and airbag use), and emergency department disposition variables were additionally extracted to characterize patient and clinical context potentially associated with imaging utilization. Temporal trends in WBCT utilization were also examined among groups stratified by Injury Severity Score (ISS). Patients were grouped into four cohorts according to their ISS (1–8, 9–14, 16–24, >24) based on established literature [12].

Categorical and continuous variables are reported as proportions (%) and medians with interquartile range (IQR), respectively. Pearson's χ^2^ was used to compare categorical variables while the adjusted Wald test was used to analyze continuous variables. Given the large sample in the study, effect size was further examined using standardized mean differences (SMD), with significance defined at levels >0.1 [13]. Multivariable logistic regression models controlling for age, sex, race, insurance coverage, injury severity score, and initial heart rate were utilized to determine the independent association of regional severe injury, state trauma center level, and hospital ownership with rates of WBCT utilization when compared to SCT. Covariates were selected based on clinical and sociodemographic relevance and prior literature [17]. An additional logistic regression model was developed using in-hospital mortality as the outcome with the same covariate set, to evaluate the association between imaging strategy and survival. Goodness of fit was evaluated using receiver-operating characteristics (ROC) and calibration plots. Logistic model outputs are reported as adjusted odds ratios (AOR) with 95% confidence interval. Temporal trends were assessed for significance using Cuzick's nonparametric test (nptrend) [14]. All statistical analyses were performed using Stata version 18.1 with statistical significance set at *α* < 0.05 (StataCorp, College Station, TX).

## Results

Among 465,977 subjects meeting inclusion criteria, 78,796 (16.9%) received WBCT, 209,468 (45.0%) SCT, and 177,713 (38.1%) did not receive a CT scan. Subsequent comparisons of imaging strategy were restricted to the 288,264 patients who underwent CT scan, among whom WBCT comprised 27.3% and SCT 72.7%. The WBCT and SCT cohorts were similar with respect to age, sex, racial distribution, rates of transfer to an operating room from the emergency department, and proportions of severe head, abdominal, or extremity injury (Table 1). However, WBCT patients were more likely to be treated at ACS-verified trauma centers (78.9 vs 68.5%, SMD = 0.24) and at for-profit hospitals (17.3 vs 11.0%, SMD = 0.18).

**Table 1:** Patient demographics and clinical characteristics: whole-body computed tomography (WBCT) vs selective computed tomography (SCT).

	SCT (n = 209,468)	WBCT (n = 78,796)	P-value	SMD
Age (years; median [IQR])	36 [26–51]	35 [26–50]	<0.001	0.04
Female (%)	33.9	32.7	<0.001	0.03
Race (%)			<0.001	0.02
White	65.5	64.4		
Black	19.6	21.8		
Asian/Pacific Islander	2.3	3.0		
Native American	1.0	0.5		
Other	11.6	10.3		
Hispanic or Latino (%)	15.8	20.1	<0.001	0.11
ISS (median [IQR])	9 [5–13]	9 [5–14]	0.27	0.01
Insurance coverage (%)			<0.001	0.01
Private	53.7	54.7		
Medicare	3.7	3.5		
Medicaid	17.6	17.0		
Uninsured	16.8	16.0		
Other	8.2	8.8		
Hospital bed size (%)			<0.001	0.03
≤200	5.2	5.8		
201–400	26.4	25.3		
401–600	33.1	31.2		
>600	35.3	37.7		
Emergency department disposition (%)			<0.001	0.01
Floor bed	40.8	39.2		
Observation unit	4.2	5.2		
Telemetry/step-down unit	9.9	11.4		
Home with services	0.1	0.1		
Deceased	0.1	0.1		
Other (jail, institutional care)	0.4	0.7		
Operating room	10.4	9.6		
Intensive care unit	15.7	15.0		
Home without services	16.8	17.3		
Left against medical advice	0.7	0.7		
Transferred to another hospital	0.9	0.7		
For-profit hospital (%)	11.0	17.3	<0.001	0.18
State Level I Trauma Center (%)	57.3	57.0	0.24	0.01
ACS verification (%)	68.5	78.9	<0.001	0.24
Transfer to OR (%)	10.4	9.6	<0.001	0.02
Severe head injury (%)	9.2	7.5	<0.001	0.06
Severe abdomen injury (%)	21.9	21.9	0.89	0.00
Severe extremity injury (%)	14.1	14.1	0.61	0.00
Illegal drug use (%)	19.7	21.7	<0.001	0.05
Alcoholism (%)	4.5	4.3	0.01	0.01
Congestive heart failure (%)	0.8	0.8	0.90	0.00
Psychiatric cond. (%)	8.6	7.7	<0.001	0.03
Hypertension (%)	16.3	15.7	<0.001	0.02
Cancer (%)	0.1	0.1	0.03	0.01
Diabetes (%)	7.6	7.4	0.17	0.01
End stage renal disease (%)	0.5	0.4	0.48	0.00
Smoker (%)	28.8	28.1	<0.001	0.02

ACS-COT Non-Verified centers maintained a substantially lower WBCT utilization throughout the study period (20.1 vs 30.2%, *p* < 0.001) (Table 2, Fig. 3). Furthermore, the unadjusted WBCT utilization decreased in both ACS-COT Verified (31.9 in 2018 to 30.2% in 2021; nptrend <0.001) and ACS-COT Non-Verified (22.0 in 2018 to 18.4% in 2021; nptrend <0.001). Incidence of WBCT and SCT both increased significantly at ACS-COT Verified centers (nptrend<0.001), while only incidence of SCT increased significantly in ACS-COT Non-Verified centers (nptrend<0.001) (Fig. 3). WBCT incidence decreased throughout the study period in non-verified centers (nptrend<0.001).

**Table 2:** Demographics and characteristics for ACS-COT non-verified vs ACS verified centers.

	ACS-COT non-verified trauma centers (n = 125,498)	ACS-COT verified trauma centers (n = 340,479)	p-value	SMD
Age (years; median [IQR])	37 [26–51]	36 [26–51]	<0.001	0.01
Female (%)	34.7	34.2	<0.001	0.01
Race (%)			<0.001	0.08
White	64.8	65.2		
Black	23.5	19.4		
Asian/Pacific Islander	2.2	2.5		
American Indian	0.6	1.0		
Other	8.9	11.9		
Hispanic or Latino (%)	9.7	18.4	<0.001	0.25
ISS (median [IQR])	8 [4–13]	9 [5–13]	<0.001	0.04
Type of CT Scan (%)			<0.001	0.18
No scan	34.2	39.6		
Selective CT	52.6	42.1		
Whole-body CT	13.2	18.3		
Transfer to OR (%)	10.9	11.3	<0.001	0.01
State Trauma Level I (%)	41.9	67.8	<0.001	0.54
Insurance coverage (%)			<0.001	0.20
Private	50.9	55.3		
Medicare	3.9	3.8		
Medicaid	14.2	18.8		
Uninsured	16.3	16.0		
Other	13.7	6.1		
Hospital bed size (%)			<0.001	0.11
≤200	3.4	5.0		
201–400	27.9	22.8		
401–600	34.6	30.3		
>600	34.1	41.9		
For-profit Hospital (%)	12.3	11.5	<0.001	0.03
Severe head injury (%)	8.4	7.6	<0.001	0.03
Severe abdomen injury (%)	22.3	22.9	<0.001	0.02
Severe extremity injury (%)	14.5	14.4	0.48	0.01
Illegal drug use (%)	16.2	19.0	0.01	0.07
Alcoholism (%)	4.1	4.4	<0.001	0.02
Congestive heart failure (%)	0.8	0.8	0.56	0.01
Psychiatric cond. (%)	7.7	8.9	<0.001	0.04
Hypertension (%)	17.3	16.8	<0.001	0.01
Cancer (%)	0.1	0.1	0.49	0.01
Diabetes (%)	7.6	7.9	<0.001	0.01
End stage renal Disease (%)	0.4	0.5	<0.001	0.02
Smoker (%)	29.9	28.5	<0.001	0.03

**Fig. 2:**
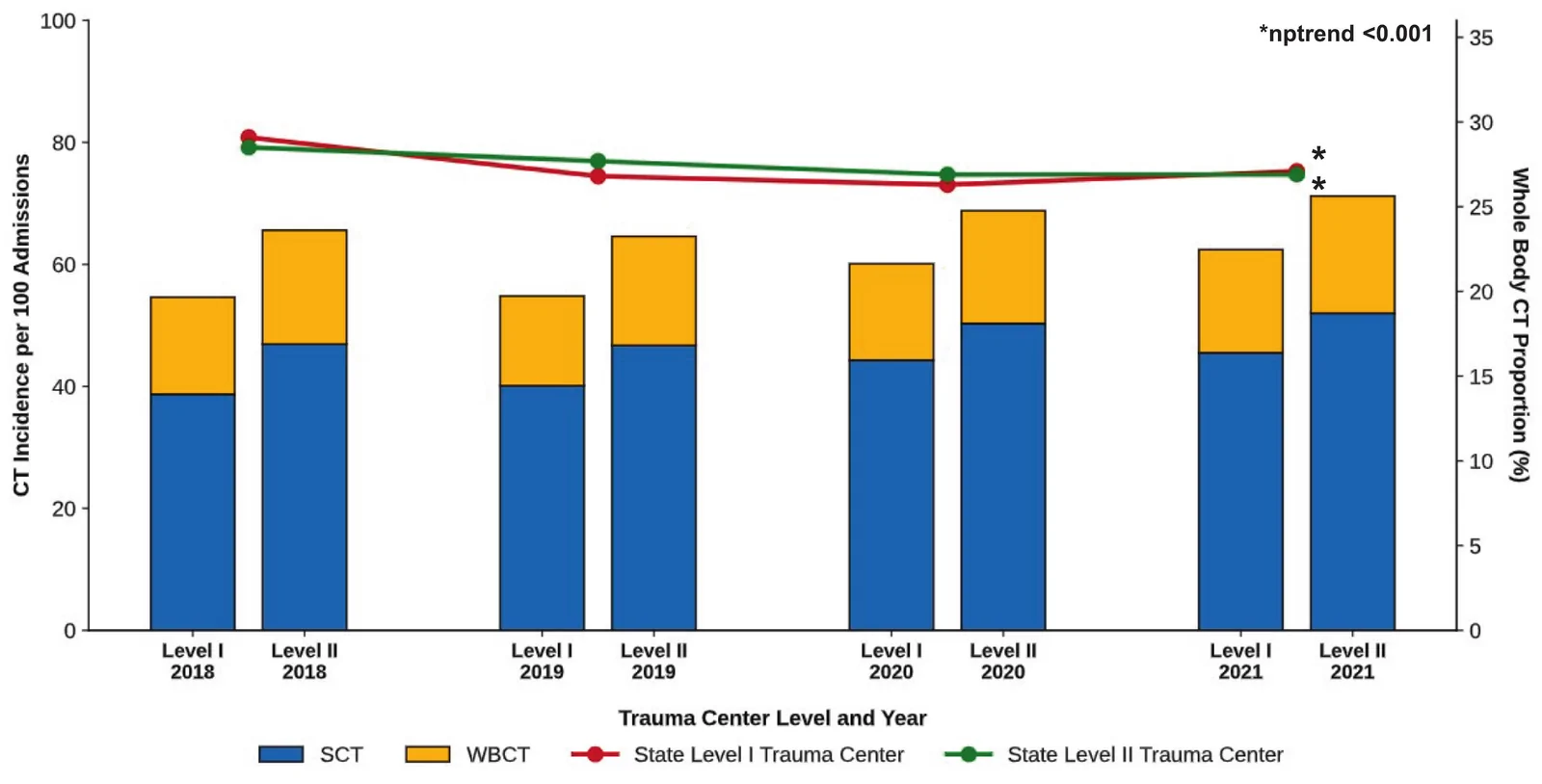
Whole-body CT utilization and incidence at state Level I and II trauma centers.

Among state-designated trauma centers, the proportional use of WBCT declined over the study period at both Level I (29.1 to 27.1%, nptrend <0.001) and Level II (28.5 to 26.9%, nptrend <0.001) facilities (Fig. 2). In contrast, Level I centers demonstrated a significant increase in overall CT use. At Level II facilities, aggregate utilization increased for both imaging strategies, with WBCT rising from 18.7 to 19.2% (nptrend <0.001) and SCT increasing from 46.9 to 52.0% (nptrend <0.001). When stratified by ISS, increasing score corresponded with a stepwise rise in WBCT use including ISS 1–8 (29.1 to 27.1%, nptrend <0.001), 9–14 (28.7 to 27.1%, nptrend <0.001), 16–24 (29.6 to 26.9%, nptrend <0.001), and >24 (29.3 to 26.1%, nptrend = 0.005).

**Fig. 3:**
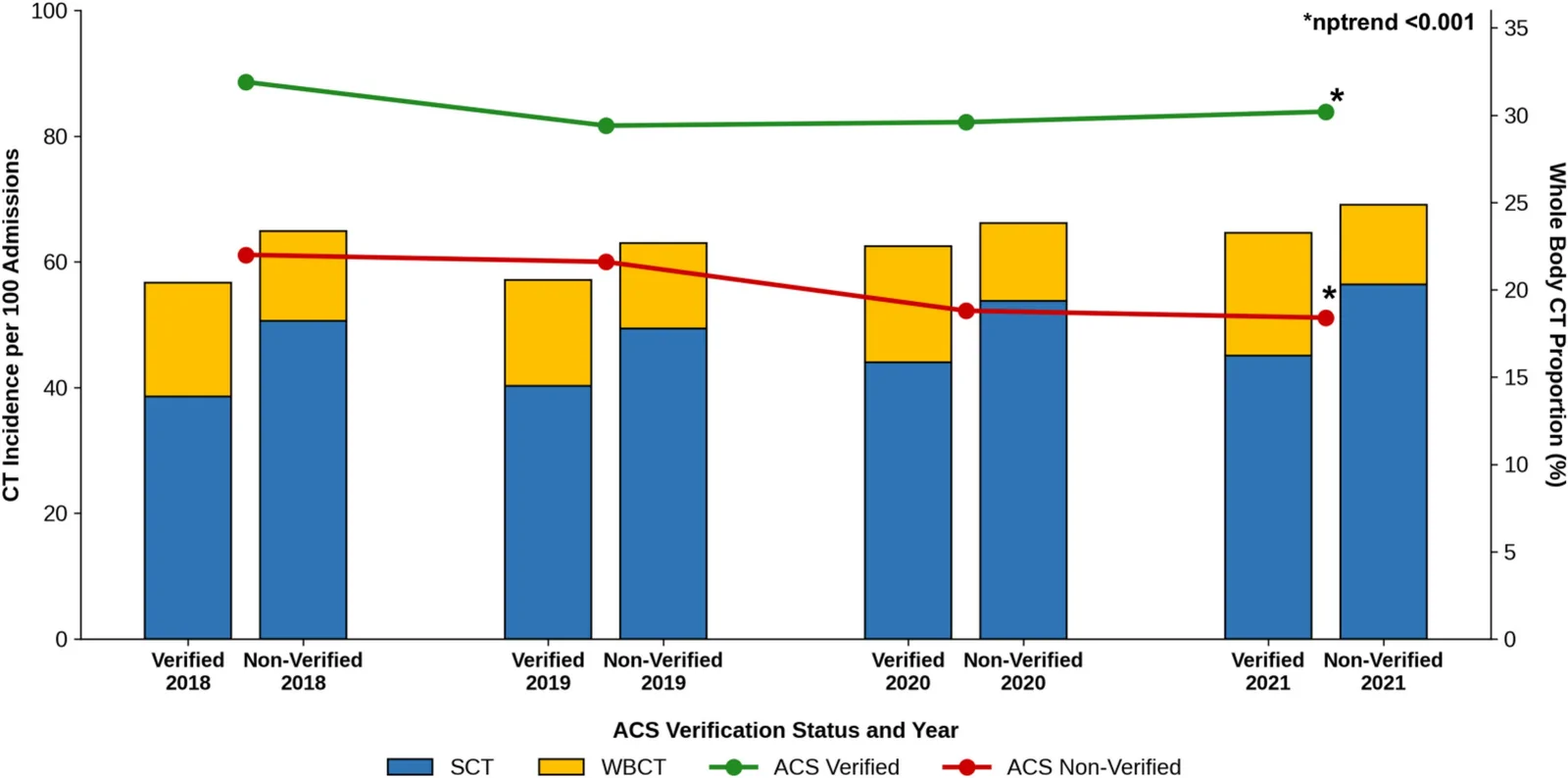
Whole-body CT utilization and incidence at ACS verified vs non-verified trauma centers.

**Fig. 4:**
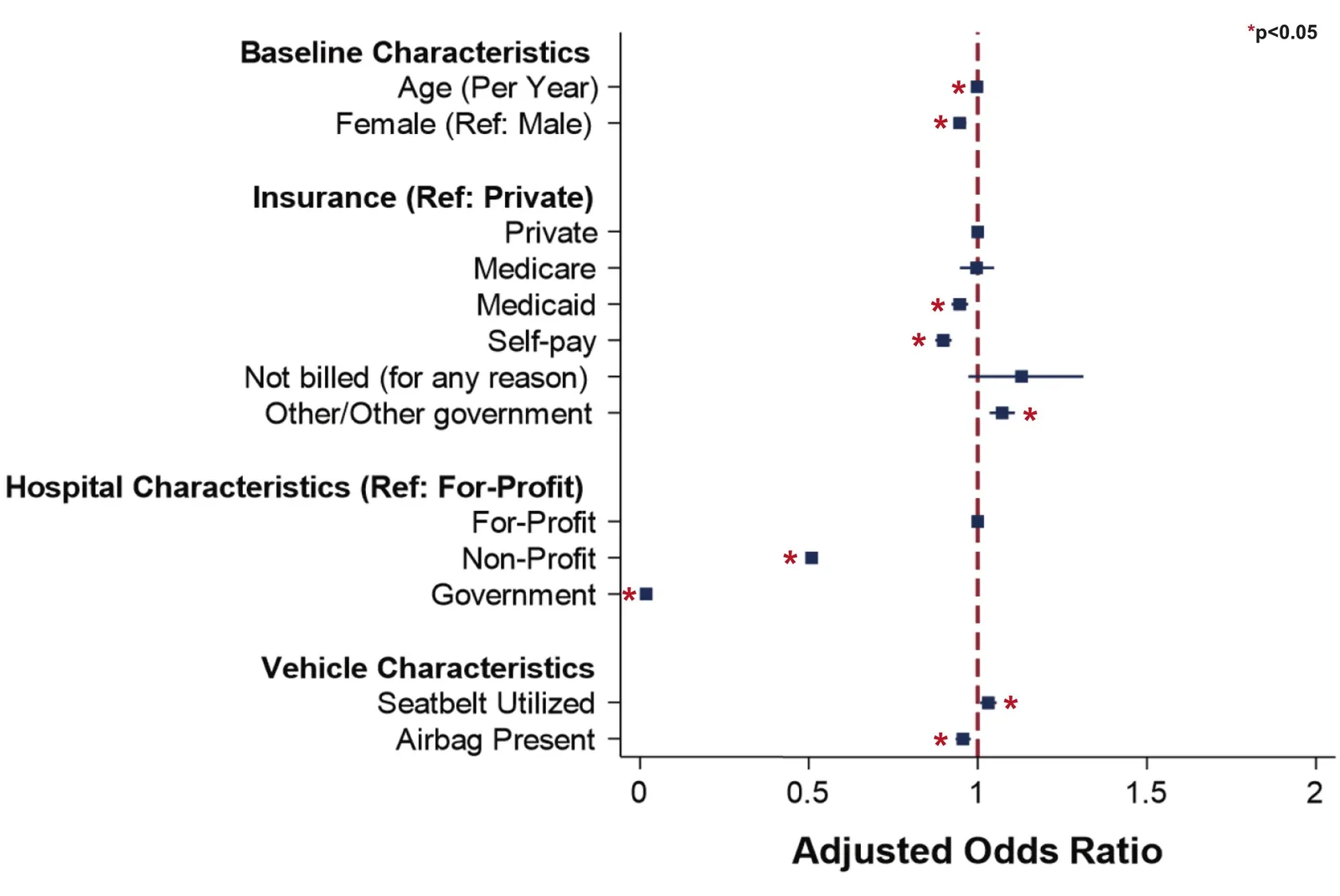
forest plot of factors associated with whole-body computed tomography utilization after multivariable adjustment.

After adequate risk adjustment, treatment at an ACS-verified center was associated with higher odds of WBCT utilization (AOR 1.96, 95% CI 1.92–2.00). In contrast, patients treated at non-profit or government hospitals were significantly less likely to undergo WBCT (AOR 0.55, 95% CI 0.54–0.57). Severe head injury was also independently associated with reduced WBCT use (AOR 0.79, 95% CI 0.76–0.81; *p* < 0.001) (Table 3). Compared with ISS 1–8, higher ISS strata were associated with higher likelihood of WBCT, including ISS 9–14 (AOR 1.05, 95% CI 1.03–1.07), 16–24 (AOR 1.12, 95% CI 1.08–1.15), and >24 (AOR 1.08, 95% CI 1.02–1.13) (Table 3).

**Table 3:** Multivariable analysis of factors associated with whole-body computed tomography utilization, with selective computed tomography as the reference.

	Odds ratio	95% CI	p-value
Age (years)	0.99	0.99–0.99	<0.001
Female	0.95	0.93–0.97	<0.001
Race			
White	–	–	–
Black	1.19	1.16–1.22	<0.001
Asian/Pacific Islander	1.28	1.21–1.35	<0.001
American Indian	0.52	0.46–0.58	<0.001
Other	0.71	0.69–0.74	<0.001
Hispanic or Latino	1.62	1.58–1.66	<0.001
Insurance coverage			
Private	–	–	–
Medicare	0.98	0.93–1.02	0.35
Medicaid	0.93	0.90–0.95	<0.001
Uninsured	0.90	0.88–0.92	<0.001
Other	1.20	1.16–1.24	<0.001
Severe head injury	0.79	0.76–0.81	<0.001
Severe abdomen injury	0.97	0.93–1.02	0.27
Severe spinal injury	0.93	0.88–0.99	0.02
Severe extremity injury	1.04	0.99–1.10	0.10
State trauma center level			
Level I	–	–	–
Level II	1.07	1.05–1.09	<0.001
ACS verification	1.96	1.92–2.00	<0.001
Hospital ownership			
For-profit	–	–	–
Non-profit/govt	0.55	0.54–0.57	<0.001
Trauma volume	0.99	0.99–0.99	0.001
Injury severity score			
1–8	–	–	–
9–14	1.05	1.03–1.08	<0.001
16–24	1.12	1.08–1.15	<0.001
>24	1.08	1.02–1.13	0.004

Importantly, non-profit (AOR, 0.51; 95% CI, 0.50–0.52) and government hospitals (AOR, 0.02; 95% CI, 0.01–0.04) were associated with reduced odds of WBCT compared to for-profit centers (Fig. 4). Other factors associated with increased odds of receiving a WBCT are displayed in Table 3 and included Hispanic ethnicity (AOR, 1.62; 95% CI, 1.58–1.67), Black race (AOR, 1.21; 95% CI, 1.18–1.24), Asian/Pacific Islander race (AOR, 1.28; 95% CI, 1.20–1.35), and seatbelt utilization (AOR, 1.03; 95% CI, 1.01–1.06). Factors associated with decreased odds of WBCT also included Medicaid insurance (AOR, 0.95; 95% CI, 0.92–0.97), uninsured status (AOR, 0.90; 95% CI, 0.87–0.92) and airbag utilization (AOR, 0.96; 95% CI, 0.93–0.98). Additionally, in a separate risk-adjusted analysis, WBCT was not significantly associated with in-hospital mortality compared to SCT (AOR 0.87, 95% CI, 0.71–1.06).

## Discussion

The present study utilized a nationally representative trauma cohort to evaluate recent trends in whole-body computed tomography (WBCT) among hemodynamically and neurologically stable patients after motor vehicle collisions. We found that while the proportional utilization of WBCT declined between 2018 and 2021, the absolute volume of CT scans increased. In addition, ACS verification and for-profit ownership were independently associated with greater odds of WBCT use. These findings highlight evolving patterns in trauma imaging. Several of these findings warrant further discussion.

The observed decline in WBCT utilization over time suggests a shift toward more selective imaging in the evaluation of stable trauma patients. Notably, this change occurred despite increasing trauma volumes and greater patient complexity, which suggests a deliberate recalibration of diagnostic strategies rather than reduced clinical need. Concurrent advances in selective CT techniques, improved image resolution, and broader adoption of evidence-informed imaging guidelines may have increased confidence in targeted scanning approaches [15], [16]. In parallel, emerging evidence, including the REACT-2 trial evaluating the mortality benefit of routine WBCT among stable patients, may have further reinforced efforts to balance diagnostic yield against potentially avoidable radiation exposure [4], [17]. Although the absolute number of WBCT studies has risen with expanding trauma volume, the declining proportional use is consistent with institutional movement toward more judicious imaging pathways. Nonetheless, substantial practice variation remains, as national data continue to demonstrate liberal WBCT use among patients with low injury severity at many centers [18], [19]. Collectively, these trends may also reflect improved bedside assessment, expanded point-of-care ultrasound use, and more refined selection criteria for comprehensive cross-sectional imaging.

These findings both extend and diverge from prior national work. In a Trauma Quality Improvement Program analysis spanning 2007 to 2016, Bunn and colleagues reported rising WBCT utilization that was concentrated at Level II centers [17]. In our contemporary cohort the proportional trend has instead declined, suggesting an inflection in imaging practice over the past decade. Our analysis further separates state-designated trauma level from ACS-COT verification status and adds hospital ownership, and verification and ownership emerged as the institutional factors most strongly associated with WBCT. The Level II effect was directionally concordant with Bunn but substantially attenuated once these factors were considered. In contrast to that utilization-focused work, the absence of a mortality difference between WBCT and SCT suggests that greater imaging intensity did not confer a survival benefit.

Beyond these temporal and outcome comparisons, the institutional gradient itself warrants closer attention, and ACS-COT verification showed the strongest association with WBCT in our cohort. Because this association persisted after adjustment for injury severity and physiology, it is unlikely to reflect patient acuity alone, though unmeasured differences in case complexity cannot be fully excluded. Verification standards require immediate and continuously available cross-sectional imaging and encourage standardized multidetector protocols for each body region, which lower the operational threshold for comprehensive scanning and may establish WBCT as a default pathway rather than a deliberate choice [20]. Verified centers also operate within a performance-benchmarking culture in which institutional metrics are routinely compared against peer programs, and qualitative work has shown that these reports shape practice and resource use, potentially discouraging centers from appearing as outliers on imaging completeness [21]. Medicolegal pressures may compound these tendencies, as a substantial proportion of trauma CT is ordered for defensive purposes despite rarely altering management, a pattern plausibly heightened at higher-visibility verified institutions [22]. These mechanisms operate without a demonstrated survival advantage for WBCT in stable patients [4], and the broader association between verification and improved outcomes remains inconsistent [23]. Taken together, the greater imaging intensity at verified centers more likely reflects protocol-driven institutional culture than evidence-based patient selection.

We further noted hospital ownership to alter imaging practices. After adjustment for demographic and injury-related factors, for-profit hospital status was associated with higher odds of WBCT use compared with non-profit and government institutions. This pattern is consistent with prior work showing greater utilization of resource-intensive services in for-profit settings, where reimbursement structures and institutional incentives may favor more comprehensive diagnostic evaluation [20]. Importantly, in our risk-adjusted analysis, WBCT was not associated with lower mortality, suggesting that more intensive imaging does not translate into a survival benefit. Together, these findings support the interpretation that elevated WBCT use at for-profit centers may be driven more by structural or economic factors than by measurable improvements in outcomes. These results underscore the need for further work evaluating the cost-effectiveness of WBCT, cumulative radiation exposure, and downstream testing and resource utilization associated with routine comprehensive imaging.

Race and ethnicity were also independently associated with WBCT utilization, and these associations warrant cautious interpretation. Among racial groups, Black and Asian or Pacific Islander patients had higher adjusted odds of WBCT relative to White patients, whereas American Indian and other patients had lower odds. Hispanic ethnicity carried the largest increase in odds of any demographic factor examined. Several non-exclusive mechanisms could contribute to this pattern. Language discordance may limit the history available at the bedside and prompt broader imaging when the mechanism or symptom burden cannot be clarified through direct communication. Disparities in clinical assessment and diagnostic trust have been described across trauma and emergency care, and where they operate they may lower the threshold for comprehensive scanning in patients perceived as more difficult to evaluate. These patient-level explanations do not readily account for the lower odds seen in American Indian and other patients, and a more consistent interpretation may lie in where different populations receive care. Groups concentrated at high-volume verified centers, which tend to be urban, would be expected to encounter protocol-driven imaging more often, whereas those treated disproportionately at lower-resource or non-verified facilities would encounter it less [21], [22]. Differences in injury pattern and physiologic presentation that the injury severity score does not fully capture may further modify these tendencies. Because TQIP does not record clinician reasoning or the granular clinical context of each imaging decision, these associations should be read as hypothesis-generating rather than as evidence of a specific causal pathway, and they identify race and ethnicity as meaningful axes of variation in trauma imaging that merit dedicated prospective study.

Severe head injury was independently associated with lower odds of WBCT, a finding that initially appears counterintuitive given that a concerning head injury might be expected to prompt broader imaging. Several features of the cohort and of trauma workflow help reconcile this result. Because eligibility required a Glasgow Coma Scale of 15, patients whose head injury manifested as depressed consciousness were not included, and the severe head injury designation in this population reflects an anatomic classification that is often established by the head CT itself rather than a clinical trigger present before imaging. In a neurologically intact patient with a localized concern, focused head CT is frequently the first and sometimes the only study obtained, and when that study demonstrates significant intracranial injury the immediate priority shifts to neurosurgical assessment and definitive management. Completion of chest and abdominal imaging within the two-hour window used to define WBCT may then be deferred or forgone as care is expedited, which would reduce the likelihood of a qualifying whole-body study. An isolated head injury in an otherwise stable patient may also lack the high-energy multisystem mechanism that most reliably prompts pan-scanning, further favoring a targeted approach [23], [24]. These considerations suggest that the association reflects the sequencing and selectivity of imaging in stable head-injured patients rather than a lower overall imaging burden, and the neurologic stability criterion that defines the cohort should be regarded as a source of selection when interpreting this specific result.

Two vehicle safety variables showed associations that ran counter to initial expectation, with seatbelt use linked to slightly higher odds of WBCT and airbag deployment to slightly lower odds. Both effects were small, with adjusted odds ratios close to one, and their statistical significance reflects the size of the cohort more than a large difference in practice. Even so, each is consistent with a recognized clinical pattern. Restrained occupants can present with a seatbelt sign, the linear abdominal wall ecchymosis that marks a substantially elevated risk of intra-abdominal injury, particularly to the hollow viscus and mesentery, and this finding reliably prompts abdominal and often comprehensive imaging even when the patient appears stable [25]. Airbag deployment, although it signals a crash of sufficient force to trigger inflation, functions primarily to cushion the occupant and is associated with reduced injury severity, including a lower rate of flail chest in registry analysis, which may lower the threshold for pursuing whole-body imaging [26]. Together these mechanisms offer a plausible account of the observed directions, though the small effect sizes caution against interpreting either association as a strong determinant of imaging strategy.

Although our analysis focused on utilization patterns, prior evidence helps contextualize the clinical implications of WBCT. Across randomized and observational studies, routine WBCT for hemodynamically stable trauma patients has not demonstrated a survival benefit compared with selective CT strategies. At the same time, WBCT carries potential downsides, including higher cumulative radiation exposure and a greater likelihood of incidental findings that can prompt additional testing, prolong evaluation, or lead to low-value interventions [27]. In this context, the proportional decline in WBCT use observed here is consistent with a broader move toward evidence-informed imaging pathways that prioritize safety, efficiency, and value. However, the increasing frequency of WBCT at high-acuity centers suggests that comprehensive imaging remains appropriate when multisystem injury is suspected or when the clinical examination is limited. Ongoing improvements in adjunctive diagnostics, including extended FAST, point-of-care imaging, and rapid laboratory assessment, may further support selective imaging by improving early risk stratification and helping reserve WBCT for patients most likely to benefit.

The present work has several important limitations. The retrospective design and reliance on TQIP data restrict the level of clinical granularity available, precluding assessment of the specific indications prompting imaging, radiation dose parameters, and patient-centered outcomes such as length of stay, morbidity, and functional recovery. Additionally, although WBCT was operationalized as head, chest, and abdomen CT obtained within two hours of presentation, meaningful variation in institutional imaging pathways and implementation across centers may have introduced heterogeneity and potential misclassification. The exclusion of dedicated pelvic and cervical spine imaging from this definition may have similarly contributed to misclassification, although this constraint is shared with prior registry-based studies that adopted comparable definitions [17]. Furthermore, while our models accounted for key demographic and injury-related factors, residual confounding is possible, as prehospital care, EMS triage protocols, transfer patterns, and clinician-level decision-making are not fully captured in TQIP and may influence both imaging selection and outcomes.

Several features of the study population also warrant caution. Participation in the Trauma Quality Improvement Program is mandatory for ACS-verified centers but voluntary for non-verified hospitals. Non-verified centers that elect to submit data may be better resourced and more attentive to quality metrics than those that do not participate, which could compress the observed difference in WBCT utilization between the two groups. The verification effect reported here may therefore understate the true association. Because the cohort was limited to adults aged 18 to 65, the findings may not generalize to patients older than 65, who represent a substantial and growing share of motor vehicle collision presentations and whose imaging and outcomes may follow different patterns. The cohort was also restricted to hemodynamically and neurologically stable patients, in whom baseline mortality is low and the absolute number of deaths was small. The risk-adjusted analysis may therefore have been underpowered to detect a modest survival difference between imaging strategies, and the absence of a mortality difference is better interpreted as the lack of a detectable benefit in this low-risk population than as evidence that WBCT confers no survival advantage in trauma more broadly. The study period overlaps the COVID-19 pandemic, which altered trauma volumes and imaging practice during 2020 and 2021 and may confound the observed temporal trends. A sensitivity analysis restricted to the pre-pandemic period was considered, but the two remaining years provide too short an interval to characterize a temporal trend reliably, and the pandemic is therefore acknowledged as a potential confounder rather than addressed through exclusion. Nonetheless, the large, nationally representative cohort and rigorous analytic approach strengthen the credibility of these findings and provide contemporary insight into evolving WBCT utilization patterns.

In conclusion, the proportional use of WBCT among stable trauma patients declined between 2018 and 2021, while overall incidence increased across both ACS-verified and state-designated centers. These divergent trends likely reflect the interplay of an expanding evidence base, evolving accreditation and quality benchmarks, and health system level incentives that shape diagnostic practice. Moreover, the absence of a significant mortality difference between WBCT and SCT recipients suggests that greater imaging intensity alone does not translate into improved survival, underscoring the importance of aligning imaging pathways with value-based care. Future work should prioritize identifying the clinical scenarios and patient subgroups most likely to benefit from WBCT, and should rigorously evaluate cost-effectiveness, radiation exposure, and downstream utilization to inform evidence-based, high-value trauma imaging strategies.

## CRediT authorship contribution statement

**Daniel Tabibian:** Writing – review & editing, Writing – original draft, Visualization, Software, Methodology, Investigation, Formal analysis, Data curation, Conceptualization. **Jeffrey Balian:** Writing – review & editing, Validation, Supervision, Software, Methodology, Investigation, Data curation, Conceptualization. **Kevin Tabibian:** Writing – review & editing, Methodology, Conceptualization. **Arjun Chaturvedi:** Writing – review & editing, Visualization, Methodology, Investigation, Formal analysis, Conceptualization. **Barzin Badiee:** Writing – review & editing, Methodology. **Troy Coaston:** Methodology, Formal analysis, Conceptualization. **Nam Yong Cho:** Visualization, Validation, Software, Methodology. **Areti Tillou:** Validation, Supervision. **Peyman Benharash:** Validation, Supervision.

## Declaration of competing interest

The authors declare that no external funding was received for this work. Dr. Peyman Benharash discloses being a proctor for Atricure; however, this relationship is unrelated to the present study.
