# Supplementary material for: Utilization of whole-body computed tomography in trauma: The role of trauma center level and ACS verification
Source: Surg Open Sci. 2026 Sep 2;34:9–16. doi: 10.1016/j.sopen.2026.08.003 (PMC13580070; doi:10.1016/j.sopen.2026.08.003)
Supplement: Supplementary file 1 [file mmc1.docx]

**Supplemental Table 1.** Demographics and Clinical Characteristics of State Level I vs Level II Trauma Centers.

|  | ***State Level I***  ***Trauma Centers***  (n = 283,430) | ***State Level II Trauma Centers***  (n = 182,547) | ***p-value*** | **SMD** |
| --- | --- | --- | --- | --- |
| Age (years; median [IQR]) | 36 [26-51] | 37 [26-51] | <0.001 | 0.02 |
| Female (%) | 34.5 | 34.1 | 0.02 | 0.01 |
| Race (%) |  |  | <0.001 | 0.03 |
| White | 63.2 | 68.0 |  |  |
| Black | 23.7 | 15.5 |  |  |
| Asian/Pacific Islander | 2.3 | 2.7 |  |  |
| American Indian | 0.8 | 1.0 |  |  |
| Other | 10.0 | 12.8 |  |  |
| Hispanic or Latino (%) | 15.3 | 17.1 | <0.001 | 0.05 |
| ISS (median [IQR]) | 9 [5-14] | 6 [4-12] | <0.001 | 0.16 |
| Type of CT Scan (%) |  |  | <0.001 | 0.18 |
| No scan | 41.8 | 32.4 |  |  |
| Selective CT | 42.3 | 49.0 |  |  |
| Whole-body CT | 15.9 | 18.6 |  |  |
| Transfer to OR (%) | 12.2 | 9.6 | <0.001 | 0.08 |
| ACS Verification (%) | 81.4 | 60.1 | <0.001 | 0.48 |
| ***Insurance coverage (%)*** |  |  | <0.001 | 0.03 |
| Private | 52.6 | 56.3 |  |  |
| Medicare | 4.0 | 3.7 |  |  |
| Medicaid | 18.7 | 15.8 |  |  |
| Uninsured | 16.7 | 15.1 |  |  |
| Other | 8.0 | 9.1 |  |  |
| ***Hospital Bed Size (%)*** |  |  | <0.001 | 0.93 |
| ≤200 | 3.0 | 7.0 |  |  |
| 201-400 | 9.7 | 46.6 |  |  |
| 401-600 | 34.7 | 26.4 |  |  |
| >600 | 52.6 | 19.9 |  |  |
| For-profit Hospital (%) | 4.7 | 22.7 | <0.001 | 0.54 |
| Severe Head Injury (%) | 7.9 | 7.6 | <0.001 | 0.01 |
| Severe Abdomen Injury (%) | 23.9 | 21.0 | <0.001 | 0.07 |
| Severe Extremity Injury (%) | 15.3 | 13.1 | <0.001 | 0.06 |
| Illegal Drug Use (%) | 18.1 | 18.4 | 0.01 | 0.01 |
| Alcoholism (%) | 4.4 | 4.2 | <0.001 | 0.01 |
| Congestive Heart Failure (%) | 0.8 | 0.8 | 0.01 | 0.01 |
| Psychiatric Cond. (%) | 9.2 | 7.7 | <0.001 | 0.05 |
| Hypertension (%) | 17.6 | 15.9 | <0.001 | 0.05 |
| Cancer (%) | 0.1 | 0.1 | 0.003 | 0.01 |
| Diabetes (%) | 8.0 | 7.4 | <0.001 | 0.02 |
| End Stage Renal Disease (%) | 0.5 | 0.4 | 0.01 | 0.01 |
| Smoker (%) | 30.4 | 26.3 | <0.001 | 0.09 |

**Supplemental Table 2.** Multivariable Analysis of Risk-Adjusted Odds of Whole-Body Computed Tomography Utilization at ACS-Verified Centers, With Selective Computed Tomography as the Reference.

|  | ***Odds Ratio*** | ***95% CI*** | ***p-value*** |
| --- | --- | --- | --- |
| Age (years) | 0.99 | 0.99 – 0.99 | <0.001 |
| Female | 0.96 | 0.94 – 0.98 | <0.001 |
| **Insurance coverage** |  |  |  |
| Private | -- | -- |  |
| Medicare | 0.89 | 0.85 – 0.94 | <0.001 |
| Medicaid | 0.92 | 0.90 – 0.95 | <0.001 |
| Uninsured | 0.92 | 0.90 – 0.95 | <0.001 |
| Other | 1.02 | 0.98 – 1.06 | 0.40 |
| Severe head injury | 0.76 | 0.73 – 0.79 | <0.001 |
| Severe abdomen injury | 0.93 | 0.88 – 0.98 | 0.01 |
| Severe spinal injury | 0.94 | 0.88 – 1.01 | 0.10 |
| Severe extremity injury | 1.07 | 1.01 – 1.13 | 0.05 |
| State Trauma Center Level |  |  |  |
| Level I | -- | -- | -- |
| Level II | 1.23 | 1.21 – 1.26 | <0.001 |
| ***Hospital Ownership*** |  |  |  |
| For-Profit | -- | -- | -- |
| Non-Profit/Govt | 0.75 | 0.73 – 0.77 | <0.001 |

**Supplemental Table 3.** Multivariable Analysis Evaluating Risk Adjusted Odds of WBCT with Selective CT as reference, at ACS Non-Verified Centers

|  | ***Odds Ratio*** | ***95% CI*** | ***p-value*** |
| --- | --- | --- | --- |
| Age (years) | 0.99 | 0.99 – 0.99 | <0.001 |
| Female | 0.91 | 0.87 – 0.95 | <0.001 |
| **Insurance coverage** |  |  |  |
| Private | -- | -- | -- |
| Medicare | 1.24 | 1.13 – 1.37 | <0.001 |
| Medicaid | 0.88 | 0.83 – 0.93 | <0.001 |
| Uninsured | 0.79 | 0.75 – 0.84 | <0.001 |
| Other | 1.50 | 1.43 – 1.58 | <0.001 |
| Severe head injury | 0.89 | 0.83 – 0.95 | 0.001 |
| Severe abdomen injury | 1.12 | 1.01 – 1.23 | 0.05 |
| Severe spinal injury | 0.91 | 0.80 – 1.04 | 0.16 |
| Severe extremity injury | 0.96 | 0.87 – 1.07 | 0.49 |
| State Trauma Center Level |  |  |  |
| Level I | -- | -- | -- |
| Level II | 0.75 | 0.72 – 0.78 | <0.001 |
| ***Hospital Ownership*** |  |  |  |
| For-Profit | -- | -- | -- |
| Non-Profit/Govt | 0.29 | 0.27 – 0.30 | <0.001 |
